# Clonal diversity and estimation of relative clone age: application to agrobiodiversity of yam (*Dioscorea rotundata*)

**DOI:** 10.1186/1471-2229-13-178

**Published:** 2013-11-13

**Authors:** Nora Scarcelli, Marie Couderc, Mohamed N Baco, Janvier Egah, Yves Vigouroux

**Affiliations:** 1 UMR DIADE, Team DYNADIV, Institut de Recherche pour le Développement (IRD), 911 Avenue Agropolis, BP 64501, 34394 Montpellier Cedex 5, France; 2 Faculté d’Agronomie, Université de Parakou, BP 123 Parakou, Bénin

**Keywords:** Approximate Bayesian Computation, Clone, Diversity, Mutation, Yam

## Abstract

**Background:**

Clonal propagation is a particular reproductive system found in both the plant and animal kingdoms, from human parasites to clonally propagated crops. Clonal diversity provides information about plant and animal evolutionary history, i.e. how clones spread, or the age of a particular clone. In plants, this could provide valuable information about agrobiodiversity dynamics and more broadly about the evolutionary history of a particular crop. We studied the evolutionary history of yam, *Dioscorea rotundata*. In Africa, Yam is cultivated by tuber clonal propagation.

**Results:**

We used 12 microsatellite markers to identify intra-clonal diversity in yam varieties. We then used this diversity to assess the relative ages of clones. Using simulations, we assessed how Approximate Bayesian Computation could use clonal diversity to estimate the age of a clone depending on the size of the sample, the number of independent samples and the number of markers. We then applied this approach to our particular dataset and showed that the relative ages of varieties could be estimated, and that each variety could be ranked by age.

**Conclusions:**

We give a first estimation of clone age in an approximate Bayesian framework. However the precise estimation of clone age depends on the precision of the mutation rate. We provide useful information on agrobiodiversity dynamics and suggest recurrent creation of varietal diversity in a clonally propagated crop.

## Background

Asexual propagation is common among plants, fungi, microorganisms and in a limited number of animals that reproduce by parthenogenesis [1]. Asexual propagation produces descendants that are genetically identical to their parents. Mankind has taken advantage of this property for the cultivation of annual crops (cassava, potatoes, yam, taro, sweet potato, etc.), perennial plants (grapevine, olive, palms, citrus, apple, peach, cherry, etc.) and ornamental plants (lily, tulip, crocus, narcissus, etc.). Thanks to the absence of recombination, crops that reproduce by asexual propagation evolve mainly through mutation and drift. It is therefore possible to study the spread of varieties by analyzing the genetic diversity of the plants. Using this property, Moncada et al. [2] analyzed the origin and the dispersal of the “Cabernet Sauvignon” grapevine variety using 84 nuclear microsatellites. Their results suggest that France was the center of origin of this variety, which subsequently spread to Australia, Chile, Hungary, Italy, Spain and the USA, where new genotypes appeared.

Because mutations accumulate over time, the number of mutations provides information about the age of a particular clone. It is theoretically possible to use this information to obtain insights into crop evolutionary history. To our knowledge, no study has used this information to make such an inference in a vegetatively propagated crop.

Most evolutionary models were developed for organisms with sexual reproduction. The recent development of new tools [3] and of a new statistical approach [Approximate Bayesian Computation ABC, 4] will facilitate the evolutionary study of vegetatively propagated crops by providing powerful modeling methods. ABC is a simulation based method that allows a Bayesian estimation of parameters and the comparison of models [4]. In this method, data are simulated using a predefined evolutionary model for which parameters are randomly chosen in an *a priori* distribution. Summary statistics are calculated on the simulated data and compared to the summary statistics calculated on the real dataset. Using the distance between the summary statistics calculated on simulated data and on real data, an *a posteriori* distribution of the parameters can be built. ABC is currently widely used to test scenarios and to estimate parameters including divergence time and population size in plants, animals and pathogens e.g. [5-9].

Thanks to these new tools, it is now possible to go beyond the description of the genetic diversity of asexual organisms and to advance our knowledge of their evolutionary history. In this paper, we used yam, a vegetatively propagated crop, as a model to study the age of varieties.

Yam (*Dioscorea rotundata*) is an annual crop that produces starchy tubers and is widely cultivated in tropical countries, especially in West Africa [10]. Yam is clonally propagated by farmers using fragments of tubers. This farming system is advantageous since plants with a high fitness rating can be maintained identically over the years. The domestication of yam dates back 10 000 years in West Africa [11], but the scarcity of archeological remains and of in-depth genetic data does not allow the precise date or location to be identified. Recent analyses of the diversity of yam revealed marked variability at both morphological [12,13] and at molecular level [14] but also showed that yam genetic diversity is highly structured [15]. Indeed, farmers’ management of yam varieties avoids varieties being mixed and strongly selects against off-types when the tubers to be used for the next generation are chosen [16]. As a result, varieties are well differentiated and within-variety genetic diversity is very low. It is therefore possible to consider that a yam variety is a single genotype that has evolved by accumulating mutations [15]. Knowing the mutation rate and the demographic evolution of the varieties, it should be possible to estimate when the varieties were created.

However, previous studies have shown that yam is not only cultivated by clonal reproduction. A practice named “ennoblement” has been reported in many countries and ethnic groups [17,18]. This practice consists in testing and selecting plants that grow spontaneously outside the cultivated fields. If these plants match farmers’ requirements, they are included in the cultivated pool as a new variety. Genetic studies have shown that farmers mainly select wild plants and interspecific hybrids between wild (*D. abyssinica* and *D. praehensilis*) and cultivated species, and that the ennoblement practice actually leads to the presence of hybrids and wild genotypes within the cultivated pool [19]. These studies also suggest that yam diversity is not as frozen as originally thought because the diversity created by sexual reproduction has been incorporated. However today, less than 5% of yam farmers continue to practice ennoblement and the practice usually fails to produce new varieties [17].

Our main objective was to assess the relative importance of clonality and sexuality in the evolution of yam diversity. To this end, we needed to determine if old and recent varieties coexist today in the yam gene pool. The underlying hypothesis is that a variety that has accumulated mutations must have been multiplied by clonal propagation over a long period and can thus be considered as an “old” variety. Conversely, a variety that has not accumulated mutations must have been created recently by sexual reproduction and can thus be considered as a “recent” variety.

In this study, we analyzed the genetic diversity of 820 individual yam plants belonging to seven varieties using 12 nuclear microsatellite loci. We created a model to simulate the evolution of genetic diversity by clonal reproduction. Using Approximate Bayesian Computation (ABC) methods to compare the genetic diversity observed based on real and simulated data, we then estimated the relative age of the varieties with the aim of deciphering the evolutionary dynamics of yam diversity.

## Results

### Genetic diversity of yam varieties

**Figure 1:**
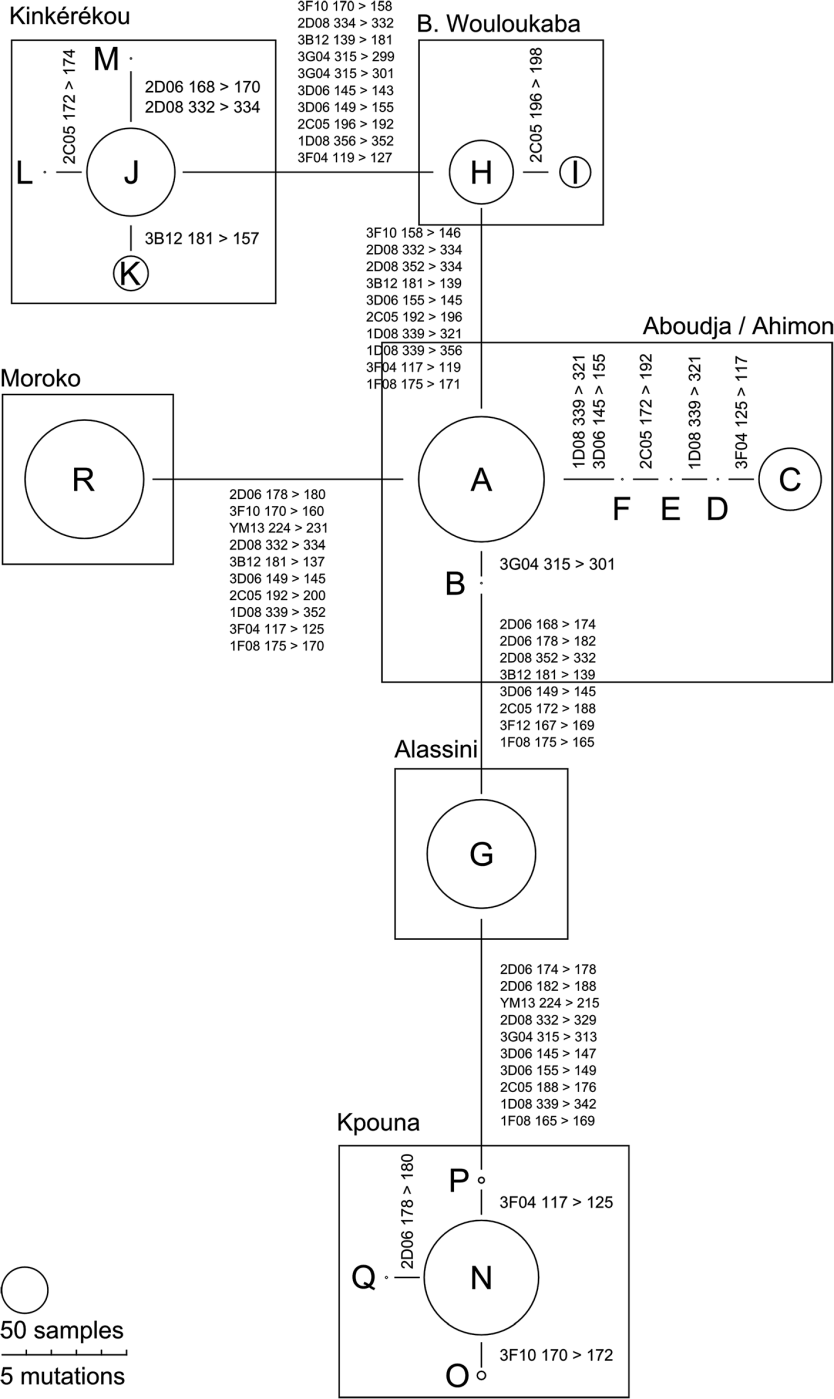
**Minimum spanning network representing the relationships between genotypes and varieties.** Each genotype is identified by a letter (see Table 1). The size of the circle is proportional to the number of corresponding samples. Relations between genotypes are represented by a bar. The size of the bar is proportional to the number of mutations between the two genotypes concerned.

The genotyping of 12 microsatellite loci on 820 samples, representing 7 varieties, revealed 18 genotypes. Table 1 shows the distribution of genotypes within varieties. A mean of three genotypes was observed per variety (minimum: 1; maximum: 5). Except for two varieties (Aboudja and Ahimon), the genotypes were variety specific. The relationships among genotypes are presented in Figure 1. Except for the same two varieties (Aboudja and Ahimon), the genotypes specific to each variety were clearly differentiated with a minimum of eight mutations. Within varieties, the genotypes were closely related, with a maximum of two mutations. The varieties Ahimon and Aboudja shared genotypes A and C separated by five mutations, but in different proportions: 94% of Aboudja samples were genotype A while 70% of Ahimon samples were genotype C. With the information at our disposal, it was impossible to decide between a common origin and mixing of the two varieties.

**Table 1:** Distribution of genotypes among varieties and farmers

		**Name of genotype**																	
**Name of variety**	**Farmer ID**	**A**	**B**	**C**	**D**	**E**	**F**	**G**	**H**	**I**	**J**	**K**	**L**	**M**	**N**	**O**	**P**	**Q**	**R**
Aboudja	BNB	28		2			1												
	OSA	82		5															
Ahimon	AS	7		22		1													
	BY			26	1														
	ONK	16	1	11															
Alassini	BNB							33											
	LBi							44											
	SA							39											
B. Wouloukaba	LM								24	10									
	LBa								34										
	WSO								10	23									
Kinkérékou	AG										27	3	1	1					
	AGa										33								
	BY										30	3							
	LBa										3	31							
Kpouna	OI														30				
	OM														20	9	1	3	
	SA														23		6		
	SY														49				
Moroko	LM																		30
	LBa																		30
	SA																		34
	WSO																		33

### Simulated data - test of the method

**Figure 2:**
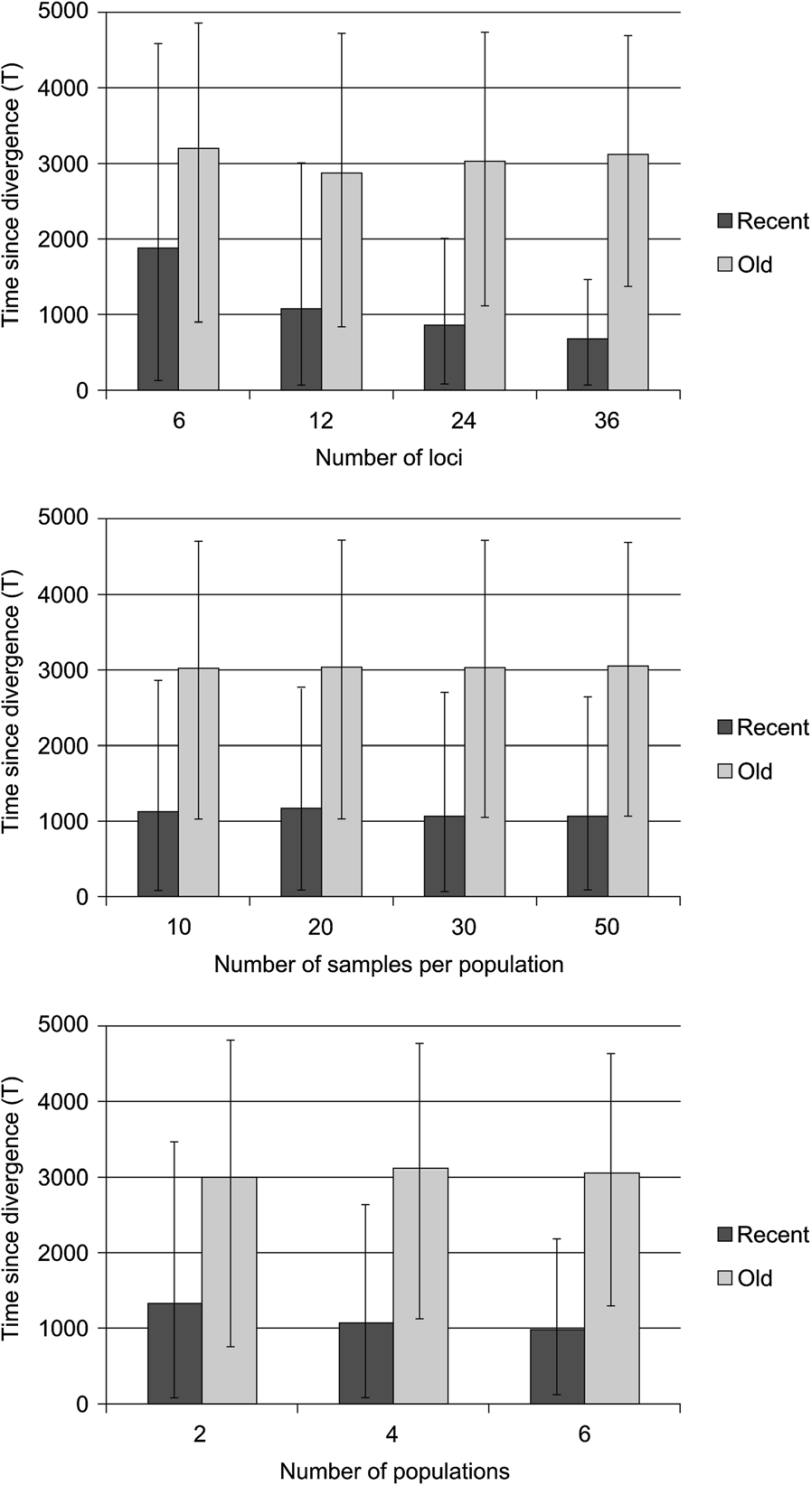
**Estimation of T for the artificial varieties according to the number of loci, the number of populations, and the number of samples per population chosen to simulate the data.** Histograms represent the median. Bars represent the 95% quantiles upper and lower bounds. Expected values are T = 500 for recent varieties and T = 3000 for old varieties.

Estimations of the age of artificial varieties are shown in Figure 2. Detailed results can be found in the supplementary data file (Additional file 1: Figure S1, Additional file 1: Table S1 and Additional file 1: Table S2).

The method was tested using simulated datasets (Figure 2). We first confirmed significant correlations between the statistics used (K_k_, Nb_k_, Fst_k_k’_ and CLst_k_k’_) and parameter T, ranging from 0.071 (p < 0.05) to 0.479 (p < 0.0001). This suggests that the statistics used are sufficiently informative to be used for the estimation of T. Detailed results for each simulation model can be found in the supplementary data file.

Overall, the method was able to correctly estimate the age of the simulated varieties (Figure 2). Across the 48 simulations, the mean age of the simulated old varieties was estimated at 3055 generations (95% CI 1072–4726) while the expected age was 3000. The age of simulated recent varieties was always overestimated with a mean age of 1128 (95% CI 109–2750) while the expected age was 500 generations. We observed that the estimated age and the confidence interval of recent varieties was highly dependent on the number of loci used. The number of populations sampled also affected results for recent varieties but to a lesser extent, while the number of samples per population had no impact on the estimation. Estimations of the age of old varieties were not strongly affected by the number of loci and the sampling.

### Yam data - correlations between summary statistics and parameters

Correlations between summary statistics (K, Nb, Fst and CLst) and parameter T were estimated using 1000 randomly selected simulations (Table 2). Like when we tested the method, the correlations between summary statistics and parameters were significant.

**Table 2:** Correlations between statistics and parameter T

	**μ**			
	**0.0005**	**0.0001**	**0.00005**	**0.00001**
K_k_	0.029	0.135***	0.183***	0.129***
Nb_k_	0.218***	0.002	0.034	0.187***
CLst_k_k’_	0	0.026	0.084**	0.166***
Fst_k_k’_	0.224***	0.351***	0.365***	0.171***

Correlation between statistics (K_k_, Nb_k_, Fst_k_k’_ and CLst_k_k’_) and parameter T are presented according to the mutation rate selected (μ). Results of Student’s t-test **p < 0.01; ***p < 0.001.

Each simulation provides some summary statistics, including the number of observed genotypes per sub-population (Nb). Using 1000 randomly selected simulations per mutation rate, we calculated the mean number of observed genotypes per sub-population for each mutation rate. The mutation rate 0.00001 fitted the observed data best with a mean of three genotypes per sub-population (9 for μ = 0.00005; 14 for μ = 0.0001; 25 for μ = 0.0005).

### Estimating the age of yam varieties

**Figure 3:**
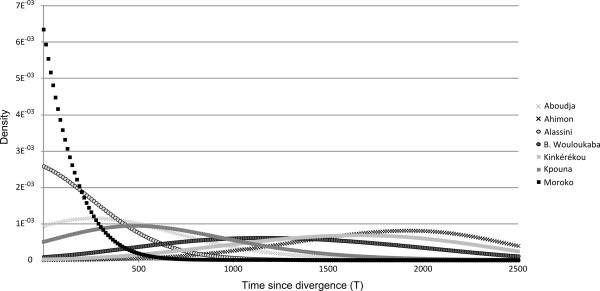
Estimation of T for the seven yam varieties (μ = 0.00001).

**Figure 4:**
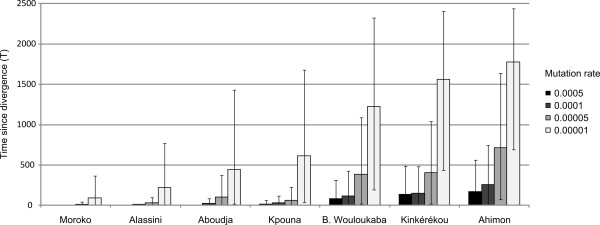
**Estimation of T for the seven yam varieties according to the mutation rate chosen for the simulations.** Histograms represent the median and bars represent the 95% quantiles upper and lower bounds.

Using this method, we estimated the age of the yam varieties using μ = 0.00001 (Table 3, Figure 3). Estimations of the ages of the varieties ranged from 93 to 1776 generations, which, considering that in the case of annual propagation, there is one generation each year, means 93 to 1776 years. The estimations of T were all associated with a large variance, but the difference between the age of the varieties was always significant (Kolmogorov-Smirnov tests, p < 0.00001). Note that the estimations of the ages of the varieties were strongly dependent on the mutation rate chosen (Figure 4). The mean age of the seven varieties was 448 with μ = 0.00001 (Table 3) while it was 112 years with μ = 0.0005. Decreasing the mutation rate by 50 therefore increases the estimated age by 7. Comparing the estimations of T for the same mutation rate allowed varieties to be ranked from the most recent to the oldest. The order was always the same whatever the mutation rate used: Moroko was the youngest variety, followed by Alassini, Aboudja, Kpouna, Bonniwouré Wouloukaba, Kinkérékou, and finally the oldest variety was Ahimon.

**Table 3:** Estimation of the age (T) of the seven varieties (μ = 0.00001)

**Name of variety**	**Median**	**95% quantile**	
		**Lower bound**	**Upper bound**
Aboudja	450	20	1431
Ahimon	1776	688	2440
Alassini	216	3	764
B. Wouloukaba	1227	192	2321
Kinkérékou	1563	435	2408
Kpouna	613	41	1681
Moroko	93	0	365

## Discussion

In this study, we analyzed the genetic diversity of seven yam varieties using nuclear microsatellite loci. Using Approximate Bayesian Computation (ABC), we showed that with our model, it was possible to estimate the relative age of the seven varieties and to classify them as old or recent. To date, this study is the first to use ABC approaches to estimate the age of clones.

### Test of the method

Using artificial varieties simulated with known parameters, we showed that our method was able to correctly estimate the divergence time. In simulations, whatever the number of populations, individuals, or loci used, high variance was always associated with the estimation of divergence time obtained for old varieties. This variance was much lower for recent varieties, especially when many loci were used. This result can be explained by homoplasy [20]. Indeed, in our model, microsatellite repeats increase or decrease randomly due to errors during the DNA replication process. The longer a variety evolves, the more mutations it will accumulate and the more homoplasy will be able to occur and consequently to scramble the evolutionary signal, leading to higher variance. Large variances are generally associated with the estimation of divergence time when the estimation is based on molecular data or on the ABC approach [8,9,21]. However, even with a large variance, it is still possible to evaluate the relative age of a variety with respect to other varieties. Here our goal was not estimate the absolute age of yam varieties but to understand if some varieties are younger than others.

Our results suggest that with this particular model, sampling at least 20 individuals per populations is a good strategy. Moreover, if possible, it is more efficient to use a few samples (10 or 20 for example) from many populations rather than a lot of samples (50 for example) from a few populations. Indeed, the T parameter is mainly correlated with the statistics Fst and CLst, two statistics based on the comparison of population genetic structure. Whatever the sampling size, the estimation was relatively good when at least 12 markers were used. More markers led to less variance but did not markedly affect the result. Our field sampling was a compromise between the number of populations sampled and the number of samples per population. We sampled at least 27 individuals per population, and when possible, we sampled as many populations as we could. The final number of populations varied from two to five, with a majority of three populations per sample. We used 12 microsatellite loci, a number associated with satisfactory estimations in our simulation study.

### Limit of the approach

Our study is one of the first to use the ABC approach to estimate the relative age of clones. However, to be well understood, we have to explain the limit of the approach. Here we used a simple model in which all populations of a given variety are created at the same time, the size of the population is fixed, and there is no migration between populations. We acknowledge that this model may not be able to fully represent the complex reality of cultivation. However, it is a useful first approach to estimate the relative age of clones.

One important parameter is the mutation rate. This was particularly apparent in this study: switching from μ = 0.0005 to μ = 0.00001 multiplied the estimated age by a factor of 7. To date, there is no estimation of the mutation rates for yam microsatellites but we know from previous studies that they can vary from 10^-2^ (human) to 10^-6^ (drosophila) depending on the organism and on the microsatellite pattern [22,23]. Here, we chose to present the result of the simulation for the mutation rate (0.00001) that best fitted the number of observed genotypes in our real dataset. Without knowing the exact mutation rate, it is still possible to estimate the relative ages of varieties, which was the purpose of this study.

Finally, our method was able to estimate when a variety split into several populations in the village in which we collected our samples. To interpret our data, we thus need to consider that the date we obtained corresponds to the divergence time of several populations from the same variety and that the original clone could be older.

### Implications of our result for yam agrobiodiversity

Like several other root and tuber crops, yam is cultivated using clonal propagation. Because of the absence of sexual reproduction, it is easy to hypothesize that such crops evolve only by mutation and drift. However, in the last 20 years, it has become clear that traditional cultivation has also used sexual reproduction, even if not by directly planting seeds but by selecting seedlings growing spontaneously in or outside the field (yam, cassava, sweet potato, taro, [19,24-27]). Such systems can be very advantageous because the best genotypes are maintained identical while new genotypes can be created thanks to sexual reproduction [19,28]. Thanks to the use of genetic markers, there is no doubt that original genotypes have been introduced into the yam germplasm by ennoblement [28]. However, we know that very few farmers continue to practice ennoblement today and that, when they do, the practice often fails to produce a plant that can be cultivated [17,29]. It was therefore impossible to know if the original genotypes found in yam germplasm were relics of a former practice or evidence for a dynamic on-going evolutionary process. In this study, we showed that old and recent varieties co-exist, meaning that farmers are still able to create new varieties via ennoblement. Our study was performed on widely used varieties [16]. Therefore, this investigation suggests that yam cultivation is a dynamic and on-going process: new genotypes are regularly introduced and become widely used varieties. Such a dynamic process might be able to introduce new varieties that are better adapted to a new biotic or abiotic environment. However, the selective advantage of new clones has yet to be demonstrated. Another important question that remains to be answered is if such a system could sustain rapid changes at a global scale.

## Conclusion

We developed and tested a model to estimate the age of clones using Approximate Bayesian Computation. This model allowed us to estimate the relative age of yam varieties and to distinguish old and recent varieties. Our investigations strongly support the hypothesis of regular introduction of new varieties in the yam agrosystem. The framework we developed can be used for any organism that reproduces asexually and should help understand the creation and spread of clones in a wide range of organisms.

## Methods

### Plant material and genotyping

A total of 820 samples were collected in one village in northern Benin in 2010. Fifteen farmers’ fields and seven varieties were sampled. Each variety was sampled in two to four different fields. Data concerning the population size of each variety was recorded (Table 4). Leaf samples were collected randomly in each field and dried with silica gel.

**Table 4:** Distribution of yam samples among varieties and farmers

**Name of variety**	**Farmer ID**	**No. of samples**	**Approximate size of variety (n.o. plants)**	**Proportion of land under each variety in the village**
Aboudja	BNB	31	100	0.8%
	OSA	87	500	
Ahimon	AS	30	200	2.5%
	BY	27	600	
	ONK	28	200	
Alassini	BNB	33	250	1%
	LBi	44	150	
	SA	39	200	
Bonniwouré Wouloukaba	LM	34	400	5.6%
	LBa	34	450	
	WSO	33	100	
Kinkérékou	AG	32	550	4.9%
	AGa	33	300	
	BY	33	350	
	LBa	34	800	
Kpouna	OI	30	200	2.1%
	OM	33	300	
	SA	29	400	
	SY	49	200	
Moroko	LM	30	450	15.8%
	LBa	30	1000	
	SA	34	1600	
	WSO	33	200	

The surface area of land used for each variety in the village was calculated based on the estimated number of plants of each variety by interviewing 15 farmers (a total of 56 varieties corresponding to 80,000 individual yam plants).

DNA extraction and genotyping were performed as described in [30]. We used 12 microsatellite loci: Dab2D06, Dpr3F10, Dab2D08, Dpr3B12, Da3G04, Dpr3D06, Dab2C05, Da1D08, Dpr3F12, Dpr3F04, Da1F08 and YM13 [31,32]. Migration was performed using an ABI Prism™ 3100 Genetic Analyzer (Applied Biosystems, Foster City, CA, USA). GeneMapper software was used to score alleles. Genotypes observed in fewer than five samples were genotyped twice (new PCR, new migration, new scoring) to ensure they were not the result of a mistake.

### Diversity analysis

For each variety, the number and the frequency of the genotypes observed were recorded. The relationships between genotypes were represented using a minimum spanning network (MSN).

### Estimation of the age of the varieties

We used an Approximate Bayesian Computation approach [33] to estimate the age of varieties by studying diversity within and between varieties.

#### Evolutionary model

A home-made program was written to simulate the evolution of populations by clonal reproduction. We used one population divided into k sub-populations evolving over T generations. N denotes the size of a sub-population. N is identical for each of the k sub-populations. After its creation, each sub-population is independent, i.e. no migration occurs between sub-populations. At T = 0, we assigned the same genotype to each individual in each population. Each generation is then created by randomly selected kxN genotypes from the previous generation. During this process, one genotype can be selected several times or can disappear due to drift. Mutations are then added to each microsatellite locus according to the mutation rate μ. The number of microsatellite repetitions increases or decreases randomly. At the end of the simulation, n samples are selected in each sub-population and summary statistics are calculated. The program is set to perform X independent simulations. Parameters n and μ are fixed at the beginning of the simulations, i.e. they are the same for X simulations. Parameters N and T are drawn from a discrete uniform distribution for each simulation.

#### Summary statistics

At the end of X simulations, four summary statistics are calculated to describe the genetic diversity and structure of the population: K_k_ is the mean number of alleles in each sub-population; Nb_k_ is the number of genotypes in each sub-population; Fst_k_k’_ is an estimation of the difference in allele frequency between two sub-populations; and CLst_k_k’_ is the estimated difference in genotype frequency between two sub-populations. CLst is similar to Fst but is calculated with genotype frequencies rather than with allele frequencies [15].

#### Approximate Bayensian Computation (ABC)

The home-made program was used to generate X simulated data and to estimate simulated summary statistics. Observed data were used to estimate the observed summary statistics. We used the module ABCestimator from the ABCToolbox package [34] to compute the ABC estimation of the parameters N and T. The number of simulations selected to estimate the general linear model (GLM) was set to 1% of the total number of simulations (X). Other parameters were set to their default values.

Correlations between the summary statistics and the parameters were tested using a Student’s t-test. Correlations were estimated on a sub-set of 1000 randomly selected simulations. A Kolmogorov-Smirnov test was used on the 1% simulations retained to compare the estimations of the T parameter between varieties.

### Testing the method

To check if our method was powerful enough to distinguish old and recent varieties, we simulated artificial varieties with known parameters, and we estimated these parameters according to the method described above. We simulated 30 recent artificial varieties with N = 1500, T = 500 and μ = 0.00001 and 30 old artificial varieties with N = 1500, T = 3000 and μ = 0.00001. Summary statistics were calculated for each artificial variety. We then calculated the mean summary statistics for the recent and old varieties. The mean summary statistics were used to compute the ABC estimation of the parameters N and T.

Parameters of the evolutionary model were set to X = 100,000; μ = 0.00001; N = [100–5000] and T = [0–5000]. We ran 48 independent simulations to test all combinations for k = 2, k = 4, k = 6, n = 10, n = 20, n = 30, n = 50 and for four different numbers of loci (6, 12, 24 and 36).

### Application to yam varieties

Parameters of the evolutionary model were set to X = 250,000; N = [100–5000] and T = [0–2500]. The mutation rate was set to four different values: μ = 0.0005, μ = 0.0001, μ = 0.00005 and μ = 0.00001, corresponding to four independent simulations sets. The number of sub-populations, k, and the number of samples selected at the end of the simulations, n, were set for each sub-population to fit the sampling (Table 4).

### Availability of supporting data

The dataset supporting the results of this article is in an additional file (Additional file 1: Table S3).

## Competing interest

The authors declare that they have no competing interests.

## Authors’ contributions

NS and YV designed and performed the research, analyzed the data and wrote the paper. NS, MC, JE and MNB performed research. All the authors read and approved the final manuscript.

## Supplementary Material

Additional file 1: Figure S1 Estimation of the time since divergence (T) for artificial varieties. **Table S1.** Correlation between statistics and parameter T for artificial varieties. **Table S2.** Estimation of the time since divergence (T) for artificial varieties. **Table S3.** Alleles observed in each genotype. Click here for file
