# Supplementary material for: Clonal diversity and estimation of relative clone age: application to agrobiodiversity of yam (Dioscorea rotundata)
Source: BMC Plant Biol. 2013 Nov 13;13:178. doi: 10.1186/1471-2229-13-178 (PMC3832681; doi:10.1186/1471-2229-13-178)
Supplement: Additional file 1: Figure S1 — Estimation of the time since divergence (T) for artificial varieties. Table S1. Correlation between statistics and parameter T for artificial varieties. Table S2. Estimation of the time since divergence (T) for artificial varieties. Table S3. Alleles observed in each genotype. [file 1471-2229-13-178-S1.pdf]

Supplementary data file

Figure S1. Estimation of the time since divergence (T) for artificial varieties.

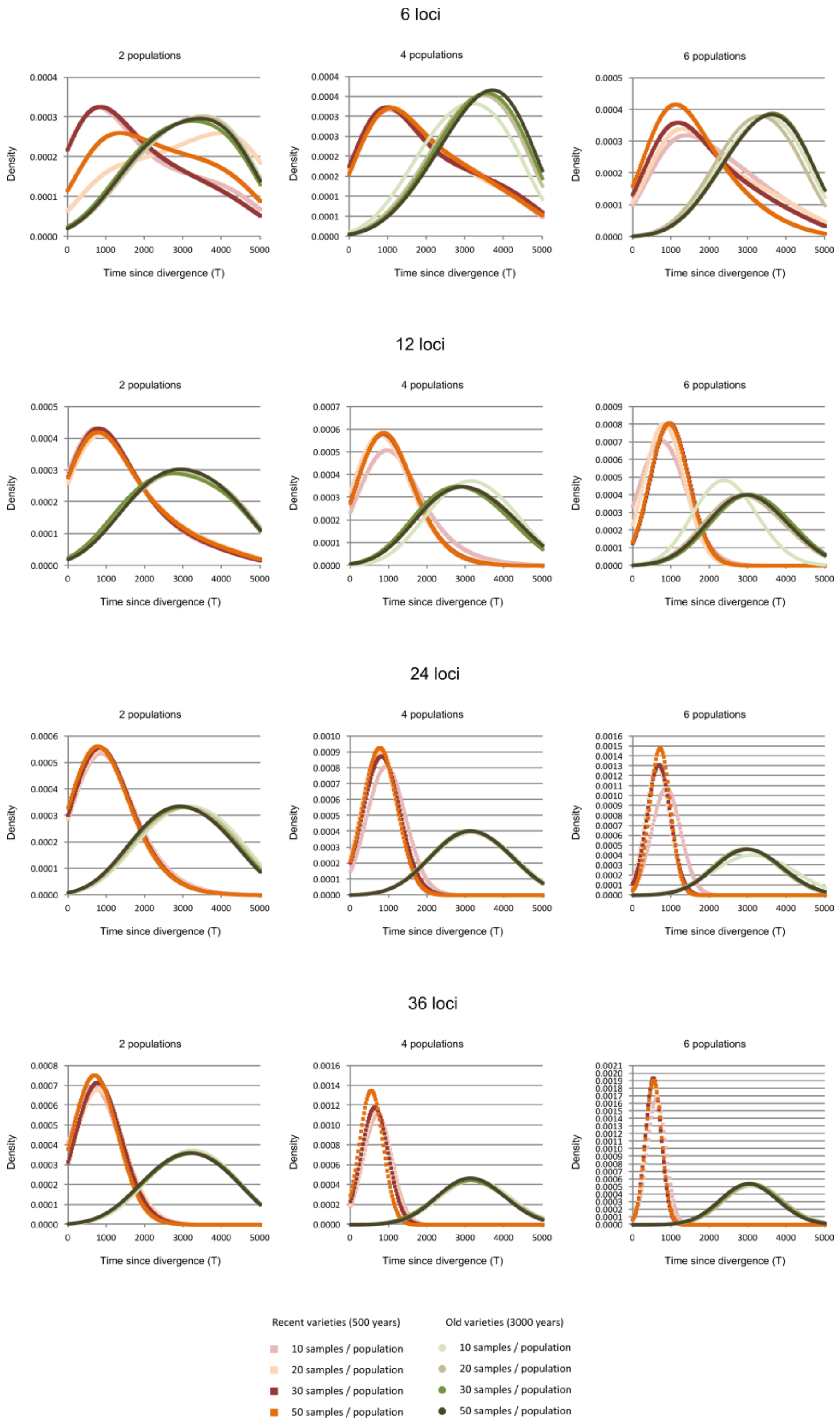

Table S1. Correlation between statistics and parameter T for artificial varieties.

	6 loci			12 loci			24 loci			36 loci		
	2 pop	4 pop	6 pop	2 pop	4 pop	6 pop	2 pop	4 pop	6 pop	2 pop	4 pop	6 pop
Nb	0.072	0.079	0.079	0.108	0.110	0.111	0.116	0.109	0.104	0.071	0.079	0.080
K	0.072	0.127	0.141	0.175	0.206	0.190	0.136	0.156	0.148	0.093	0.130	0.123
CLst	0.197	0.195	0.194	0.224	0.222	0.221	0.186	0.153	0.155	0.107	0.102	0.102
Fst	0.144	0.133	0.135	0.308	0.301	0.310	0.372	0.371	0.377	0.451	0.469	0.479

p<0.05

p<0.01

p<0.001

Table S2. Estimation of the time since divergence (T) for artificial varieties.

Recent varieties (500 years)

6 loci	2 populations				4 populations				6 populations			
	10 samples	20 samples	30 samples	50 samples	10 samples	20 samples	30 samples	50 samples	10 samples	20 samples	30 samples	50 samples
Median	1708.56	2934.23	1680.07	2210.76	1786.35	1752.42	1754.5	1786.52	1940	1827.72	1686.58	1465.77
Lower 95 quantile	96.6772	300.761	97.2559	179.748	125.926	125.759	119.896	133.463	199.243	182.527	152.556	128.129
Upper 95 quantile	4656.93	4866.75	4581.94	4740.63	4574.42	4603.65	4629.22	4592.42	4563.1	4564.16	4444.41	3978.74

12 loci	2 populations				4 populations				6 populations			
	10 samples	20 samples	30 samples	50 samples	10 samples	20 samples	30 samples	50 samples	10 samples	20 samples	30 samples	50 samples
Median	1248.71	1333.71	1266.36	1298.14	1171.8	987.682	1021.78	1022.54	853.043	856.7	981.909	967.46
Lower 95 quantile	69.5178	79.7116	73.4822	74.5618	86.8245	61.3063	71.944	73.3418	58.4779	83.7042	140.944	133.139
Upper 95 quantile	4075.73	4225.04	4086.88	4179.88	3286.69	2812.11	2694.5	2657.99	2071.75	1874.4	1986.95	1976.72

24 loci	2 populations				4 populations				6 populations			
	10 samples	20 samples	30 samples	50 samples	10 samples	20 samples	30 samples	50 samples	10 samples	20 samples	30 samples	50 samples
Median	1075.13	1053.27	1030.23	1000.91	949.232	778.293	832.38	787.19	873.248	649.282	682.172	711.128
Lower 95 quantile	70.5601	69.3383	66.5968	60.5021	123.585	77.9041	92.6446	87.8272	186.576	107.412	128.202	196.028
Upper 95 quantile	2986.82	2900.42	2839.75	2821.71	1938.85	1663.69	1746.11	1645.54	1602.49	1243.38	1277.93	1239.09

36 loci	2 populations				4 populations				6 populations			
	10 samples	20 samples	30 samples	50 samples	10 samples	20 samples	30 samples	50 samples	10 samples	20 samples	30 samples	50 samples
Median	808.888	886.586	859.725	786.42	714.593	676.601	644.111	551.733	616.075	535.256	534.926	542.803
Lower 95 quantile	45.7155	62.2837	62.5947	50.5091	97.6451	88.2556	77.6022	61.8542	159.926	134.469	142.109	143.658
Upper 95 quantile	2086.18	2160.58	2048.15	1925.9	1421.65	1356.39	1308.46	1131.59	1084.86	947.501	936.963	951.649

Old varieties (3000 years)

6 loci	2 populations				4 populations				6 populations			
	10 samples	20 samples	30 samples	50 samples	10 samples	20 samples	30 samples	50 samples	10 samples	20 samples	30 samples	50 samples
Median	3100.28	3076.37	2964.57	3036.6	3007.58	3221.51	3292.6	3374.1	3296.71	3193.03	3398.02	3382.47
Lower 95 quantile	638.825	626.447	560.951	614.152	776.5	937.882	1006.63	1049.52	1161.04	1136.3	1287.83	1261.78
Upper 95 quantile	4846.15	4840.56	4824.88	4835.71	4775.7	4825.37	4844.54	4860.72	4825.83	4789.21	4847.64	4847.47

12 loci	2 populations				4 populations				6 populations			
	10 samples	20 samples	30 samples	50 samples	10 samples	20 samples	30 samples	50 samples	10 samples	20 samples	30 samples	50 samples
Median	2828.84	2897.74	2795.67	2889.27	3099.61	2945.83	2835.03	2935.02	2403.78	2878.44	3045.56	2964.68
Lower 95 quantile	547.342	586.683	528.436	618.073	1127.53	879.535	820.727	884.805	875.226	1049.77	1186.75	1111.87
Upper 95 quantile	4795.22	4812.23	4793.29	4800.83	4783.17	4760.23	4720.87	4762.77	4050.4	4655.3	4719.61	4689.12

24 loci	2 populations				4 populations				6 populations			
	10 samples	20 samples	30 samples	50 samples	10 samples	20 samples	30 samples	50 samples	10 samples	20 samples	30 samples	50 samples
Median	3065.27	2989.17	2932.1	2906.16	3139.37	3088.75	3093.45	3111.87	3111.87	2941.66	2975.95	3001.05
Lower 95 quantile	889.901	835.48	801.062	792.849	1221.01	1200.15	1247.46	1243.64	1243.64	1326.15	1357.99	1367.37
Upper 95 quantile	4809.56	4788.25	4773.03	4759.02	4764.69	4745.44	4734.61	4745.3	4745.3	4540.24	4568.79	4595.53

36 loci	2 populations				4 populations				6 populations			
	10 samples	20 samples	30 samples	50 samples	10 samples	20 samples	30 samples	50 samples	10 samples	20 samples	30 samples	50 samples
Median	3163.14	3126.53	3122.31	3098.28	3211.04	3146.91	3114.17	3127.25	3094.01	3129.5	3069.1	3021.82
Lower 95 quantile	1132.74	1067.12	1023.54	1033.76	1515.69	1425.81	1408.11	1502.57	1584.07	1700.55	1652.12	1603.39
Upper 95 quantile	4796.68	4794.91	4796.6	4788.47	4728.02	4708.46	4690.66	4657.66	4557.09	4537.78	4482.57	4449.66

Table S3. Alleles observed for each genotype.

Genotype name	Allele size (bp)																								N.o. genotype
	Locus 2D06		Locus 3F10		Locus YM13		Locus 2D08		Locus 3B12		Locus 3G04		Locus 3D06		Locus 2C05		Locus 1D08		Locus 3F12		Locus 3F04		Locus 1F08		
	Allele 1	Allele 2	Allele 1	Allele 2	Allele 1	Allele 2	Allele 1	Allele 2	Allele 1	Allele 2	Allele 1	Allele 2	Allele 1	Allele 2	Allele 1	Allele 2	Allele 1	Allele 2	Allele 1	Allele 2	Allele 1	Allele 2	Allele 1	Allele 2	
A	168	178	158	170	224	224	332	352	137	181	315	315	149	155	172	192	339	339	167	169	117	125	175	175	133
B	168	178	158	170	224	224	332	352	137	181	301	315	149	155	172	192	339	339	167	169	117	125	175	175	1
C	168	178	158	170	224	224	332	352	137	181	315	315	145	149	192	192	321	321	167	169	117	117	175	175	66
D	168	178	158	170	224	224	332	352	137	181	315	315	145	149	192	192	321	321	167	169	117	125	175	175	1
E	168	178	158	170	224	224	332	352	137	181	315	315	145	149	192	192	321	339	167	169	117	125	175	175	1
F	168	178	158	170	224	224	332	352	137	181	315	315	145	149	172	192	321	339	167	169	117	125	175	175	1
G	174	182	158	170	224	224	332	332	137	139	301	315	145	155	188	192	339	339	169	169	117	125	165	175	116
H	168	178	146	170	224	224	334	334	137	139	315	315	145	149	172	196	321	356	167	169	119	125	171	175	68
I	168	178	146	170	224	224	334	334	137	139	315	315	145	149	172	198	321	356	167	169	119	125	171	175	33
J	168	178	146	158	224	224	332	334	137	181	299	301	143	155	172	192	321	352	167	169	125	127	171	175	93
K	168	178	146	158	224	224	332	334	137	157	299	301	143	155	172	192	321	352	167	169	125	127	171	175	37
L	168	178	146	158	224	224	332	334	137	181	299	301	143	155	174	192	321	352	167	169	125	127	171	175	1
M	170	178	146	158	224	224	334	334	137	181	299	301	143	155	172	192	321	352	167	169	125	127	171	175	1
N	178	188	158	170	215	224	329	332	137	139	301	313	147	149	176	192	339	342	169	169	125	125	169	175	122
O	178	188	158	172	215	224	329	332	137	139	301	313	147	149	176	192	339	342	169	169	125	125	169	175	9
P	178	188	158	170	215	224	329	332	137	139	301	313	147	149	176	192	339	342	169	169	117	125	169	175	7
Q	180	188	158	170	215	224	329	332	137	139	301	313	147	149	176	192	339	342	169	169	125	125	169	175	3
R	168	180	158	160	224	231	334	352	137	137	315	315	145	155	172	200	339	352	167	169	125	125	170	175	127
